# Genomic diversity and macroecology of the crop wild relatives of domesticated pea

**DOI:** 10.1038/s41598-017-17623-4

**Published:** 2017-12-12

**Authors:** Petr Smýkal, Iveta Hradilová, Oldřich Trněný, Jan Brus, Abhishek Rathore, Michael Bariotakis, Roma Rani Das, Debjyoti Bhattacharyya, Christopher Richards, Clarice J. Coyne, Stergios Pirintsos

**Affiliations:** 1 Department of Botany, Palacký University in Olomouc, Olomouc, Czech Republic; 2 Agricultural Research, Ltd., Troubsko, Czech Republic; 3 Department of Geoinformatics, Palacký University in Olomouc, Olomouc, Czech Republic; 4 The International Crops Research Institute for the Semi-Arid Tropics, Hyderabad, India; 5 Department of Biology and Botanical Garden, University of Crete, Heraklion, Greece; 6 Department of Life Science & Bioinformatics, Assam University, Silchar, India; 7 United States Department of Agriculture, National Laboratory for Genetic Resources Preservation, Fort Collins, USA; 8 United States Department of Agriculture, Washington State University, Pullman, Washington, USA

## Abstract

There is growing interest in the conservation and utilization of crop wild relatives (CWR) in international food security policy and research. Legumes play an important role in human health, sustainable food production, global food security, and the resilience of current agricultural systems. Pea belongs to the ancient set of cultivated plants of the Near East domestication center and remains an important crop today. Based on genome-wide analysis, *P*. *fulvum* was identified as a well-supported species, while the diversity of wild *P*. *sativum* subsp. *elatius* was structured into 5 partly geographically positioned clusters. We explored the spatial and environmental patterns of two progenitor species of domesticated pea in the Mediterranean Basin and in the Fertile Crescent in relation to the past and current climate. This study revealed that isolation by distance does not explain the genetic structure of *P*. *sativum* subsp. *elatius* in its westward expansion from its center of origin. The genetic diversity of wild pea may be driven by Miocene-Pliocene events, while the phylogenetic diversity centers may reflect Pleisto-Holocene climatic changes. These findings help set research and discussion priorities and provide geographical and ecological information for germplasm-collecting missions, as well as for the preservation of extant diversity in *ex-situ* collections.

## Introduction

Legumes represent the second most important family of crop plants after Poaceae, accounting for approximately 27% of the world’s crop production. Legumes play an important role in human health, sustainable food production, global food security, and the resilience of current agricultural systems^1,2^. There is a growing awareness of the need to ensure the global food supply^3,4^. One currently underdeveloped option for achieving this goal is a more systematic and targeted use of crop wild relatives (CWR) in crop breeding programs^5^. CWRs contain a wealth of genetically important traits due to their adaptation to a diverse range of habitats due to not having passed through the genetic bottlenecks of domestication. CWRs are increasingly recognized as a primary reserve of genetic variation, critical to maintaining agricultural productivity in the face of agricultural challenges^6,7^. CWRs play an important role in resolving fundamental questions concerning the domestication, ecological genetics and diversity of agronomically valuable variation^8–11^.

Pea is an emblematic plant in the field of biology, as it is linked to Mendel’s discovery (1866) of the laws of inheritance^12^. Pea belongs to an ancient set of cultivated plants of the Near East domestication center and is an economically important crop today^1,13,14^. Domesticated about 10,000 years ago^15–19^, pea, among other grain legumes, accompanied cereals in becoming an important dietary component of early civilizations in the Middle East and the Mediterranean^13^. The garden pea (*Pisum sativum* L.) belongs to the tribe *Fabeae*, which contains five genera, including important grain legumes: *Lathyrus* (grass pea); *Lens* (lentils); *Pisum* (peas) and *Vicia* (vetches)^1,14,20^. Two species, *P*. *fulvum* Sibth. & Sm. and *P*. *sativum* L., are most commonly recognized, the latter of which is divided into two subspecies, the domesticated pea subsp. *sativum* and the wild form, subsp. *elatius* (M. Bieb.) Asch. & Graebn.^1,14,21^. Populations of wild pea (*Pisum sativum* subsp. *elatius*) are scattered over the Mediterranean basin, while the distribution of *P*. *fulvum* is restricted to the Middle East^1,14,20^. Although worldwide pea germplasm includes approximately 98,000 accessions, only a small proportion (less than 1%) represent wild pea^22^. Pea genetic diversity held in collections was assessed using various morphological and molecular tools; however, wild material was largely underrepresented in these studies^1,14^. Comprehensive analysis of *Pisum sp*. diversity using retrotransposon markers revealed *P*. *fulvum* to be the most distant from cultivated pea, while *P*. *sativum* subsp. *elatiu*s is the closest^23–25^. Nevertheless, the diversity and distribution of wild *P*. *sativum* have not been explored to the extent of cultivated pea^24,25^.

In this research, we asked the following questions: 1) Is the genetic diversity of wild pea geographically or environmentally structured? 2) Is there evidence of hybridization between species? 3) Does the center of phylogenetic diversity for *Pisum* coincide with the genetic diversity centers of these species across the Mediterranean Basin and the Fertile Crescent? 4) How might climate chage impact the species distributions of these two species?

## Results

### The genetic structure of wild pea

**Figure 1:**
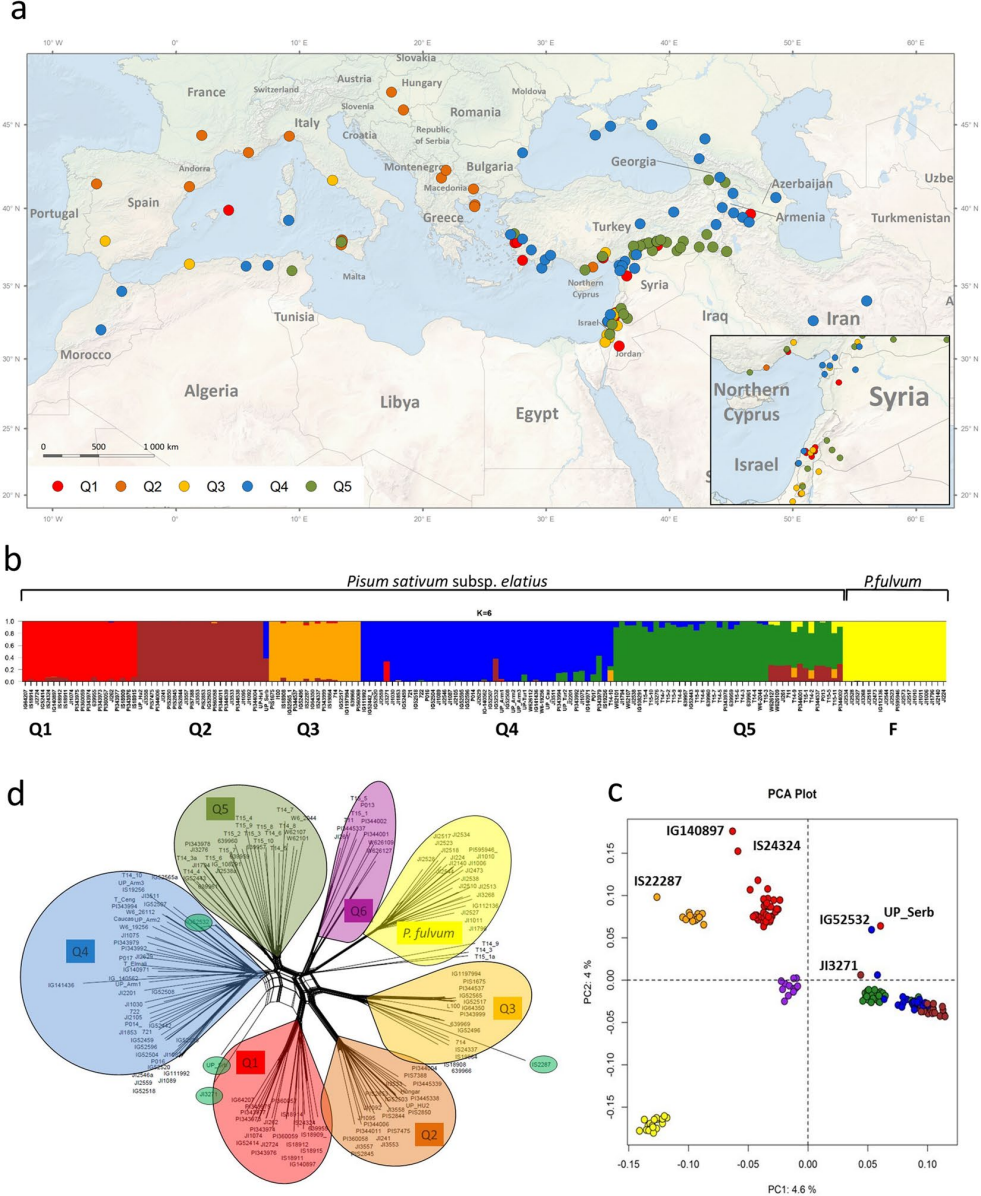
Inferred population structure of wild *Pisum sp*. based on 35,647 DArT seq SNPs. (**a**) Geographical distribution of five *P*. *sativum* subsp. *elatius* groups (ArcGIS for Desktop 10.4.1 http://desktop.arcgis.com/en). (**b**) The K value of 6 is shown, and the assignment to respective taxonomical groups is given, (**c**) Principal component analysis, coloured according to STRUCTURE groups with additional six (violet) group, (**d**) SplitsTree analysis (SplitTree v4, http://www.splitstree.org).

DArTseq analysis performed on 161 wild-origin *Pisum* samples resulted in 66,910 polymorphic markers, which, upon filtering for missing data (>10%) and minor allele frequency (MAF <  = 0.05), resulted in 35,647 SNPs, informative SNPs used for further analysis. Of these, 2,421 SNPs were mapped to the *Medicago truncatula* v 4.0 genome and were shown to be evenly distributed across the chromosomes (not shown). Allele frequency data readily resolved two groups of wild *P*. *sativum* subsp. *elatius* and the distant relative *P*. *fulvum* (Fig. 1a–d). STRUCTURE analysis revealed K = 6 to be the most probable partition of the data using the ad hoc delta K method^26^. This partioning clearly separates *P*. *fulvum* as a group from *P elatius* samples. Further analysis subdivided the *P*. *elatius* samples into 5 lineages. The five lineages vary in their genetic diversity, largely overlapping in their spatial location within the Levant, with the exception of the Q2 lineage, which showed a European location (Fig. 1a). The Q1 lineage consisted of 19 samples, 14 of which originate from Israel, Jordan and Turkey. The Q2 lineage contained 23 samples, mainly from Europe (France, Portugal, Spain, Hungary, Italy), except for two from Turkey and Israel. Q3 had 15 samples, mainly from Israel and Syria. The Q4 lineage had 43 samples of variable origin (Morocco, Georgia, Turkey, Iran and Syria, Crimea, Georgia, Armenia). The Q5 lineage had 40 samples, with 19 collected *in situ* in their origin of southeastern Turkey, and an additional 13 samples distinct at K = 7 that showed substantial admixture (0.3–0.4 membership coefficient Q).

Relative F_ST_ values supported differentiation of the *P*. *fulvum* (F) group (0.545 to 0.683), followed by the Q2 lineage (0.439 to 0.561), while weak differentiation was observed between the Q3 and Q4 *P*. *sativum* subsp. *elatius* groups in comparison to Q5 (0.139 and 0.192, respectively).

Twenty nine out of 161 DArTseq samples originated from direct *in-situ* sampling (Table S1) from eastern Europe and southeastern Turkey. We calculated a percentage of loci heterozygous per individual. In the case of *P*. *fulvum*, this percentage was 0.39% to 3.5% (mean of 0.72%); that of *P*. *sativum* subsp. *elatius* was 0.33% to 13.5% (mean of 1.48%). There were no significant differences between germplasm-derived and *in-situ* samples, although *in-situ* samples displayed a slightly higher percentage of heterozygous SNPs (0.55% to 13.5%, mean of 1.68%) than germplasm-derived samples (0.33% to 4.18%, mean of 1.45%).

However, there were outliers: *P*. *fulvum* JI2527 (3.9%) and JI1796 (1.39%), and particularly two *P*. *sativum* subsp. *elatius* samples, UP_Serbia (17.5%) and IG52532 (17.33%), followed by IG52496 (8.65%) and IG140897 (8.63%), which can be explained by their admixture status, as revealed by STRUCTURE analysis. It is worth noting that in contrast to most of the samples, UP_Serbia is a recent collection, having produced only two generations *ex situ*. Similarly, IG52532 and IG52496 were collected in Turkey back in 1988, and IG140897 from Armenia was collected in 2004 (www.genesys-pgr.org). Together, these data indicate a very low natural outcrossing rate and a high genetic homogeneity of populations.

Principal component analysis (PCA) separated *P*. *fulvum* along the first principal component and explained 4.6% of the variation, with the second principal component explaining 4% of the variation (Fig. 1c) and the four major groups of *P*. *elatius*. The ordination analysis also partitioned the *P*. *elatius* samples into multiple groups that largely correspond to the STRUCTURE partitions. Some of the known hybrid samples, such as UP_Serbia, were intermediate in the PCA bi plot (Fig. 1c).

Because the individual samples may reflect past hybrization events, a neighbor-net tree was constructed from the Hamming distance between individuals using SplitsTree software. The reticulate dendrogram is useful if the data contain incompatible signals. The incompatible or ambiguous sample placements are represented by splits with cycles or boxes, resulting in several paths between any two samples. The partitioning of the data complimented the STRUCTURE and PCA findings in that they identified a significant partition of *P*. *fulvum* from a multiply partitioned, diverse set of *P elatius* samples (Fig. 1d). The UP-Serbia, IG52532 and JI3271 samples show ambiguous placement in the splits tree network, reflecting their admixture status.

**Figure 2:**
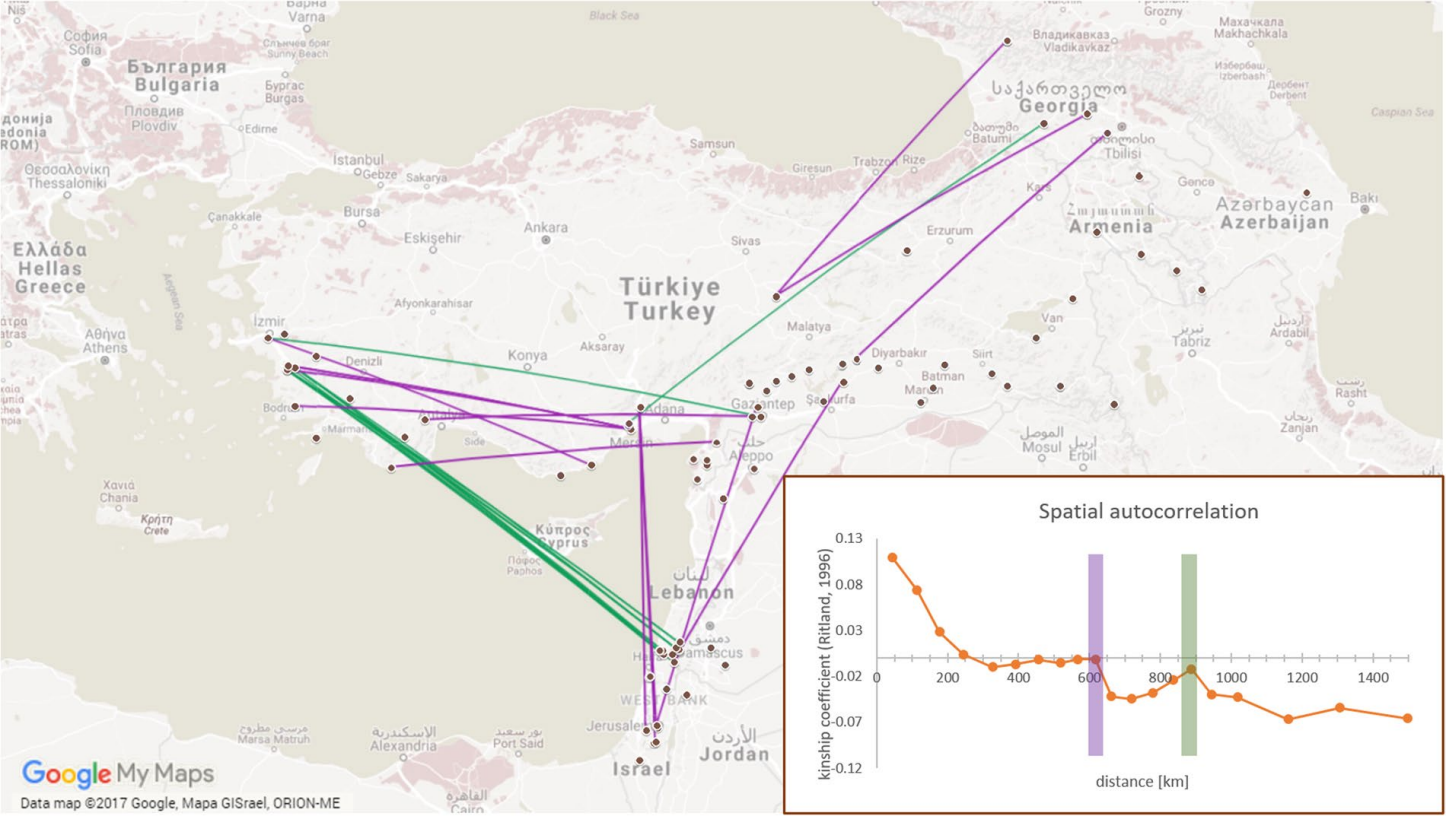
Spatial autocorrelation analysis profiles for 89 wild pea accessions from Turkey and Near East. Geographic distances on the x-axis are the means of distance classes. Bar colours (green and violet) correspond to high kinship coefficients of accessions pairwise comparisonsat 600 and 900 km peaks in inlay. (SPAGeDiver 1.5, http://ebe.ulb.ac.be/ebe/SPAGeDi.html and Google Maps (https://maps.google.com/).

We analysed the spatial distribution with a distance to centroid approach and that the clusters were mostly all overlapping and showed no isolation by distance (not shown). Furthermore we applied spatial autocorrelation analysis (Fig. 2) to *P*. *elatius* samples from Turkey and the Near East in order to minimalize the effect of wide geographical variability (mountain ranges, seas, etc.). The results indicated that kinship drops to zero at about 250 km. It is important to note, however, that the kinship realtionships beyond this distance do not monotonically decrease, but rather fluctuate in slope. This supports the hypothesis that isolation by distance does not explain all of the genetic differentiation within this species.

**Figure 3:**
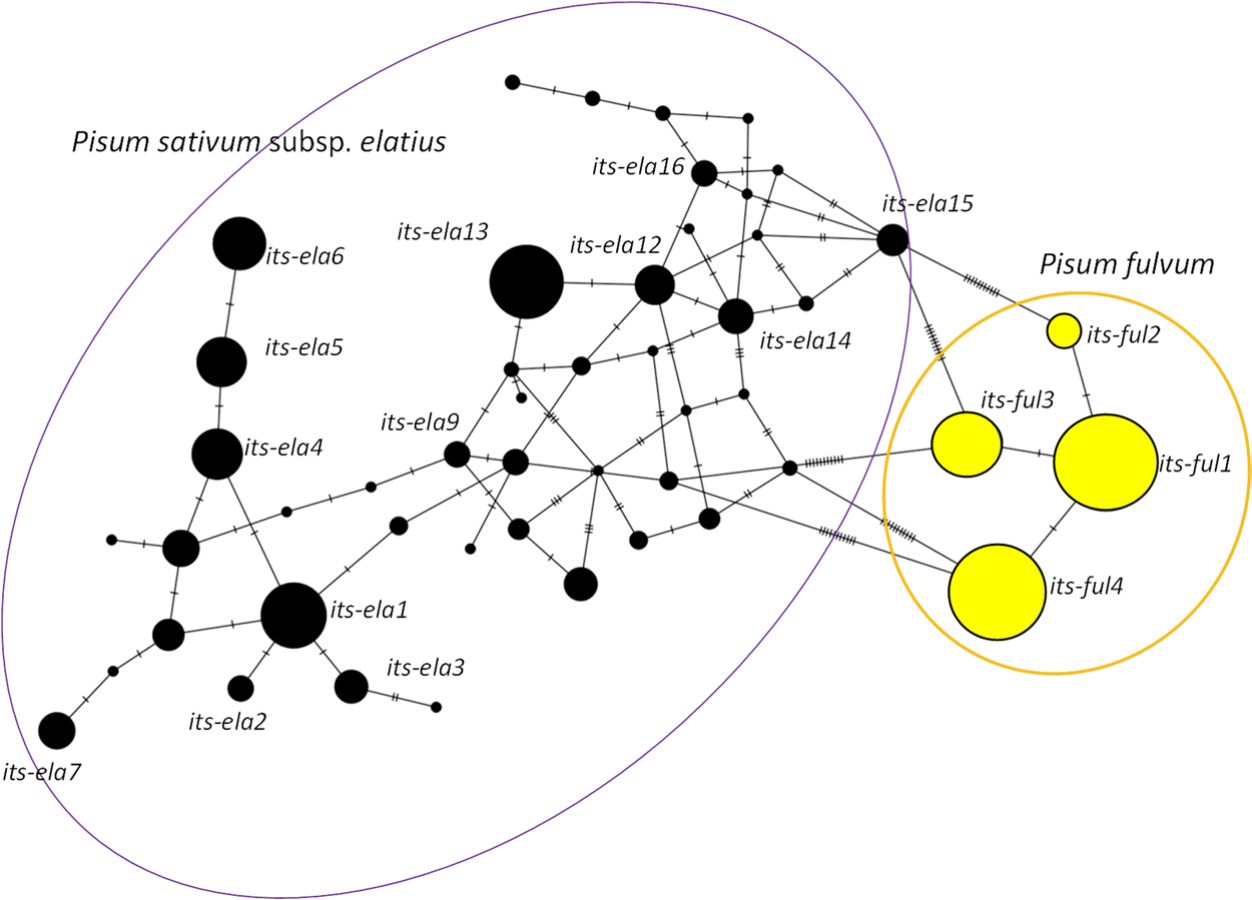
ITS network based on ITS locus with 18 polymorphic sites detecting 28 major and 17 unique haplotypes (NETWORK v5 http://www.fluxus-engineering.com/sharepub.htm#a10).

**Figure 4:**
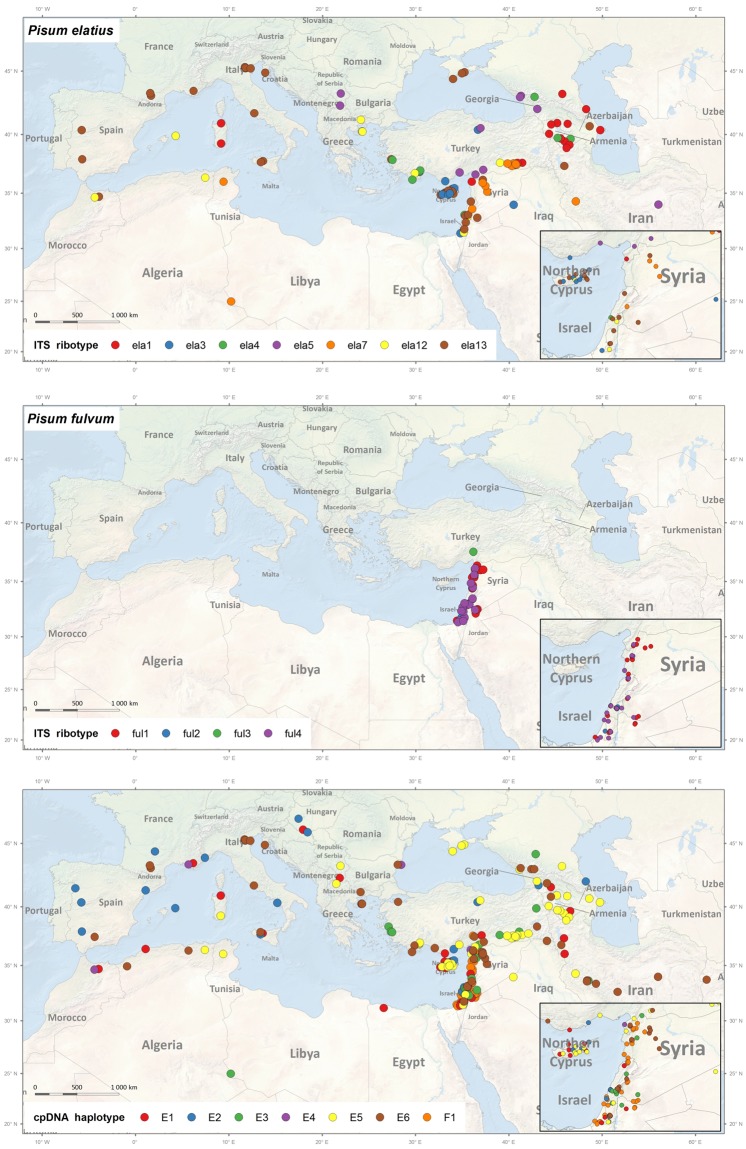
Haplotype distribution of wild pea accessions. (**a**) Of *P*. *sativum* subsp. *elatius* major ITS haplotypes, (**b**) *P*. *fulvum* ITS haplotypes, (**c**) of cpDNA *trnS-G* haplotypes (ArcGIS for Desktop 10.4.1 http://desktop.arcgis.com/en/).

As genome-wide analysis requires high-quality genomic DNA and is costly, we used a biparentally inherited ITS marker for the entire set of 364 samples. The alignment of 664 bp of ITS locus included 27 bp of 18S rDNA, 238 bp of ITS1, 164 bp of 5.8S rDNA, 213 bp of ITS2 and 22 bp of 26S rDNA, totalling 664 bp. This resulted in 18 SNPs detecting 45 haplotypes altogether (Table S1). 149 samples of *P*. *fulvum* had 4 haplotypes distinguished by one mutation step, while these haplotypes are separated from the closest “*elatius”* samples by 11 mutations (Fig. 3). The haplotypes were partly geographically structured (Fig. 4a,b), with *its-ful1* to the north (Syria, Turkey) and *its-ful3* and *its-ful4* to the south (Israel, Jordan). Wild *P*. “*elatius”* (216) samples had 24 haplotypes represented by more than one sample and 17 unique ones (Tables S1, S2). Two large and complex clusters were found, one with *its-ela1* (27) and derived (*its-ela2* to *ela8*, 69 altogether). The second cluster had *its-ela13* (41), an associated complex network of *its-ela10* to *ela24*, and unique haplotypes (63).

Similarly, uniparentally inherited chloroplast *trnS-G* locus was analyzed in a set of 364 samples (Table S1). An 855-bp region of the chloroplast *trnS-G* locus identified seven haplotypes in 364 samples, which differed by five SNPs and one six-bp indel. These defined six haplotypes in *P*. *sativum* subsp. *elatius* and one haplotype in *P*. *fulvum* (Table S1). Of the 149 *P*. *fulvum* samples, all but six had the typical *trnSG-F1* haplotype. The six exceptions were JI2510, JI2521, JI2539, VIR2523 and WL2140, which had “*elatius*” E6 and IG112136, which had the E3 haplotype (Table S1), suggesting introgression. Among the wild “*elatius”* samples, *E5* (68) and *E6* (58), followed by *E1* (34), *E2* (29) and *E3* (21), were the most abundant, while *E4* (5) was rare. *E4* was identical to *E1*, except for the six-bp (TACAAA) insertion. Geographically, *trnSG-E1* and *E6* are the most widespread, spanning the entire geographical range, while *trnSG-E3* is restricted to Turkey, Syria, Israel and the Caucasus (Fig. 4c).

Although ITS/cpDNA and DArTseq sets overlapped only partially (94/161 samples), there was no relationship between the nuclear-encoded IT, the uniparentally inherited chloroplast haplotypes, or the clusters identified by genome-wide DARTseq assignment into STRUCTURE, except for *P*. *fulvum* (Table S1).

### *P*. *fulvum* is a clearly separated species, possibly due to divergent evolution in a specific habitat

**Figure 5:**
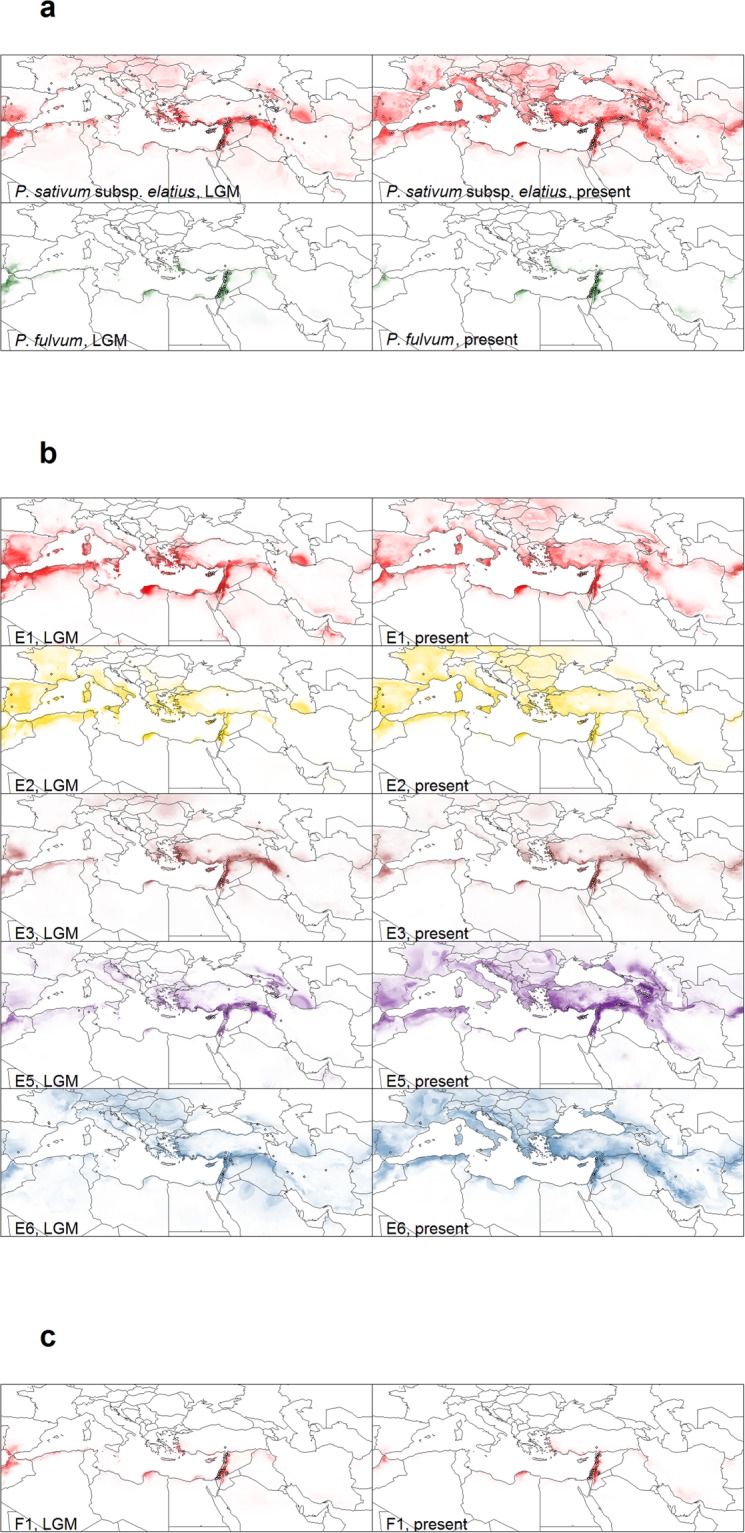
Results of ecological niche models. For (**a**) *P*. *sativum* subsp. *elatius* and *P*. *fulvum*, (**b**) haplotypes E1, E2, E3, E5 and E6 and (**c**) haplotype F1, as they resulted from sequencing analysis of cpDNA (*trnS-G*) region. Lighter colors correspond to lower probabilities of occurrence, while more saturated colors correspond to higher probabilities of occurrence (created with R version 3.2.2. https://cran.r-project.org/bin/windows/base/old/3.2.2/). White dots with black outlines represent the occurrence points that were used in the models.

**Figure 6:**
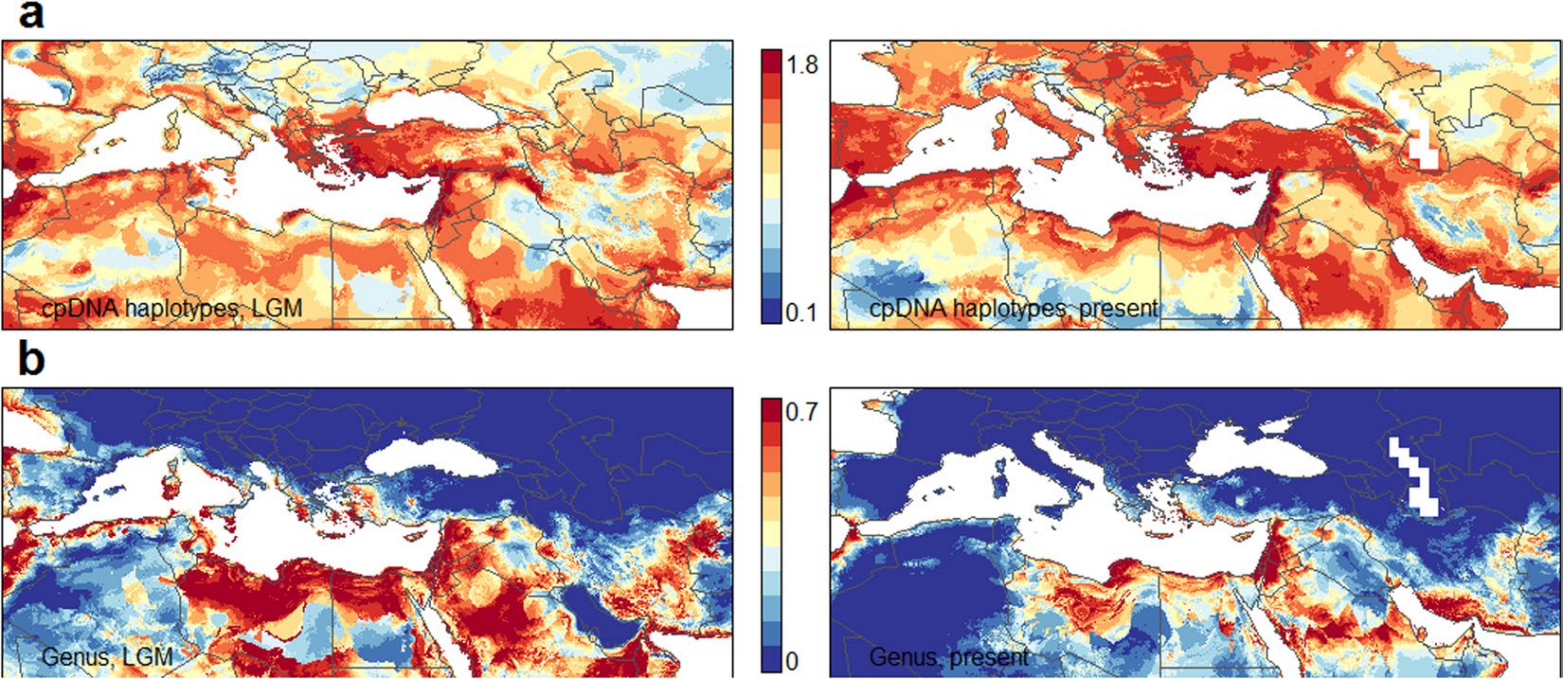
Geographical variation in Shannon’s index (**a**) of diversity, calculated from the niche model outputs for haplotypes E1, E2, E3, E5, E6 and F and (**b**) for *Pisum sativum* subsp. *elatius* and *P*. *fulvum* (created with R version 3.2.2. https://cran.r-project.org/bin/windows/base/old/3.2.2/).

Predictions of the potential current and past distribution of the two species are demonstrated by the results of niche modelling (Fig. 5a). According to model evaluations, modelling accuracy for the species *P*. *fulvum* and *P*. *sativum* subsp. *elatius* was excellent, with all Area Under the receiver operating characteristic Curve (AUC) values above 0.89. These predictions are generally in accordance with the distribution of the occurrence points, with *P*. *fulvum* showing a much narrower potential distribution than *P*. *sativum* subsp. *elatius*. In the case of *P*. *fulvum*, BIO19 (Precipitation of Coldest Quarter) had the highest permutation importance in the bioclimatic model (53.9%), followed by BIO5 (Max Temperature of Warmest Month) (18.5%) and BIO7 (Temperature Annual Range) (9.9%). For *P*. *sativum* subsp. *elatius*, BIO16 (Precipitation of Wettest Quarter) had the highest permutation importance (34.8%), followed by BIO14 (Precipitation of Driest Month) (15.5%) and BIO8 (Mean Temperature of Wettest Quarter) (15.4%). Additionally, prediction models reveal new areas of potential distribution for the two species. The outcomes of niche similarity tests are shown in Fig. S2. Spatial diversity of the niche patterns for the two wild taxa is indicated by Shannon’s index (Fig. 6b). There is a clear geographical pattern, with high diversity in the southeastern part of the Eastern Mediterranean Basin (Northern Africa, the Near East, Cyprus, the southwestern Mediterranean coasts of Turkey and the southern Aegean islands), where the spatial centers of species diversity was predicted. Of the seven cpDNA haplotypes identified, six occurred in enough locations to be modelled using Maxent (Fig. 5b,c). The model evaluation showed high predictive performance, with AUC values ranging from 0.862 ± 0.252 to 0.992 ± 0.019 (mean ± s.d.). Upon visual inspection, the spatial patterns of certain pairs, such as *trnSG*-E1 - E2, seem to be following similar patterns, while some haplotypes, such as F1, have a more distinct pattern. The tests of niche similarity (Fig. S3) offer a clearer view of the similarities between the potential niche patterns. As with the species, there was no definite case of divergence, while the pairs E1-E2 and E3-F were found to be statistically significantly similar. The spatial pattern of Shannon’s diversity index, calculated using the six cpDNA haplotypes, can be seen in Fig. 6a. The discrepancy between this pattern and the spatial diversity of the niche patterns of the two wild pea species concerns the western Mediterranean Basin and is expanded in the Balkans and the northern part of the eastern Mediterranean Basin. The projections of the distribution of the species *P*. *fulvum* and *P*. *sativum*. subsp. *elatius* and their six cpDNA haplotypes (Fig. 5a–c) during the Last Glacial Maximum (LGM) were similar compared to the current potential distribution. The pattern of Shannon’s diversity index, calculated using the projection of the distribution of the species *P*. *fulvum* and *P*. *sativum*. subsp. *elatius* during the LGM (Fig. 6b), was also similar to the current pattern of distribution and only slightly expanded on the North African part of the western Mediterranean Basin. The pattern of Shannon’s diversity index of cpDNA haplotypes (Fig. 6a), based on their predicted distribution during the LGM, was similar to the current distribution in most of the study area. Interestingly, the predicted distribution was highly impoverished in the Balkan region and the central European areas.

## Discussion

Crop wild relatives, including wild *Pisum* species, present an important source of novel, useful genetic diversity related to adaptive traits that may be of agricultural relevance^27^. While the genetic diversity of cultivated pea germplasm has been extensively analyzed over the past decade^1,14,22,24,25^, a limited number of wild pea samples has been studied^24,25,28–30^. Crop wild relatives have been extensively studied for their diversity and their genetic relationship with derived crop lineages, including legumes^31–36^. All these studies showed lower diversity of the crop compared to progenitor species. In a few cases, the gene flow between progenitor and crop was documented^32^. Our study detected a complex ITS network (Fig. 3, Table S1) more extensive than in previous studies^28,29^. Two major (*its-ela1* and *its-ela13*) *P*. *sativum* subsp. *elatius* ITS haplotypes are separated from each other (Fig. 3) but do not correspond to proposed eco-geographical or taxonomical separation^37^. Consistent with previous studies^20,23–25^, *P*. *fulvum* was identified as a clearly distinct species (Figs 1 and 3). The *P*. *fulvum* ITS haplotypes were geographically structured (not shown) into northern (Syria, Turkey) and southern (Israel, Jordan), as reported earlier^23,24^. Interestingly, six out of 146 *P*. *fulvum* accessions showed common *P*. *sativum* subsp. *elatius* cpDNA hyplotypes (E3, E6), while also having a typical *P*. *fulvum* ITS, DArTseq assignment (Table S1). This may be explained by hybridization and backcrosses, as shown in other crop-wild-relative pairs, such as *Phaseolus* and *Oryza*^38,39^. The markers studied identified several *P*. *fulvum* and *P*. *sativum* subsp. *elatius* accessions with evidence of past hybridization events^40^. The level of heterozygosity and the intermediate assignment coefficients estimated in the population structure of two *P*. *fulvum* samples (JI2527 and JI1796) suggest possible hybrid origin, perhaps a putative cross with *P*. *sativum* subsp. *elatius*. Unfortunately, the DArTseq analysis did not include *P*. *fulvum* samples displaying the cpDNA haplotype of *P*. *sativum* subsp. *elatius* (Supplementary Table S1). The situation is complex due to the possibility of rare bi-parental inheritance of the plastids^41^.

It is hypothesized that legumes differentiated sometime before the end of Cretaceous in Africa^42^, while a recent phylogenetic study, together with fossil evidence, suggested that dispersal and vicariance putatively linked to the Tethys seaway is a more likely explaination of present legume distributions^43^. The tribe Fabeae originated and evolved in the Eastern Mediterranean in the middle Miocene (23–16 Mya) and expanded thereafter^20^. The stepping-stone hypothesis^44^ has been proposed, in which shallowly submerged seamounts would emerge during the extreme glacial sea-level minima distributed between the present-day islands and the Iberian Peninsula and North Africa^45^. The long-distance dispersal events are relatively common in Fabeae^20^. This also characterizes the *Pisum* genus, which spread from its center of origin in the Middle East eastwards to the Caucasus, Iran and Afghanistan, and westwards to the Mediterranean^1,14^.

Our results on the spatial diversity of the niche patterns, as indicated by Shannon’s index, suggest that while the species´ center of diversity is in the Near East, there may be two secondary centers: (1) Northern Africa in the Eastern Mediterranean Basin and (2) the coasts of Turkey, Cyprus and the Aegean islands. These findings suggest that the Northern African route was another hitherto unreported route for the westward expansion of wild pea. Historical records also support this view. For example, Columella, an important writer of the Roman Empire, mentions that “*Roman legionaries still gathered wild peas from the sandy soils of Numidia and Palestine*, *to supplement their rations”*^46^. Numidia was the ancient region of Africa north of the Sahara, with boundaries corresponding roughly to those of modern Tunisia and Algeria. Relatively recently, it has been recognized that gradual expansion would have contributed substantially to westward or eastward colonization along the Mediterranean Basin, either across the northern (European) side or across North Africa, and have been decisive in shaping the current species and genetic diversity of the Mediterranean wild flora^47^. A gradual expansion of herbaceous Fabaceae in the opposite direction, eastward from the west, has been reported for *Anthyllis montana*^48^.

Our results indicated that the spatial structure of genetic diversity of *P*. *elatius* (cpDNA and ITS haplotypes) in their westward expansion in the Mediterranean Basin does not correlate with a strict pattern of isolation by distance^14,20^. The wild pea results are in contradiction to the diversity pattern of many species, in which there is a gradual decrease in diversity running east-west along the Mediterranean Basin^49,50^. This diversity gradient has been attributed to the role of two interrelated processes around the Pleistocene. Specifically, it has been attributed to the east-west recolonization during the Holocene and the population size contraction under local LGM climate in resident western and low-elevation populations^50^. Our results are not in agreement with this biogeographic scenario. The discordance between the predicted pea species diversity center in the southern parts of the Eastern Mediterranean Basin and the predicted genetic diversity centers, which are scattered around the Mediterranean Basin and the Balkans, agrees with the view that there is no overall correlation between genetic diversity and species diversity across the Basin^50^. In the case of pea, the absence of an east-west gradient of genetic diversity suggests a different mechanism of dispersal and colonization. Our results with pea are more closely aligned with the pattern found in Northern African populations of *Erophaca* (Leguminosae), which are much more diverse genetically than European ones, despite the plant being (currently) relatively rare in North Africa^51^.

Discordance between predicted species and genetic diversity centers of wild pea was also revealed during the LGM. This pattern is differentiated from the longitudinal decline of genetic diversity in the Mediterranean Basin^50^. This discordance seems reasonable, taking into consideration that the *Pisum* genus evolved in the Eastern Mediterranean and spread westward. During the LGM, climate was drastically harsher in the Western Mediterranean (cold and dry) compared to the more favorable climate of the Eastern Mediterranean (wetter and warmer)^47,49^. Such harsh conditions are likely to have modified the available ecological niches of several species, including pea, causing discontinuities or eliminations of the predicted species diversity centers^50^. The geographical broad projected areas of high diversity of cpDNA haplotypes during the LGM may have been facilitated by the Messinian crisis of salinity during the late Miocene. In that period, land bridges allowed for the exchange of genetic material and formulated a spatial pattern of high diversity of wild pea throughout the Mediterranean Basin. The expansion during the Messinian is in agreement with scenario concerning the colonization of the western part of the Mediterranean Basin by Irano-Turanian elements^20,52^. Our results indicated that the predicted genetic diversity centers of *Pisum* may be driven by Miocene-Pliocene events, while the predicted species diversity centers may reflect recent (Pleisto-Holocene) climatic changes.

Research on the potential effects of climate change projections on pea production is limited. However, pea production is very likely to be affected by rising CO_2_ levels and temperatures, impacting important traits such as flowering time, mycorrhizal colonization, water use and photosynthesis^53^. There is no related research done on wild pea or on the question of how plastic it can be in its natural habitat.

In future studies, biotic interactions (including endophytes) may be a critical factor in understanding both range alterations and responses to climate change in pea. Although we did not explore this interaction, evidence among other legume species such as *Medicago* suggest that their endophytic diversity impacts their colonization success. For example, it has been hypothesized that the geographic expansion of *Medicago* was directly influenced by the geographical diversity of rhizobia symbionts^54^.

These results describing the genetic diversity of wild *Pisum* and their spatial and environmental structure suggest that these important genetic resources are under pressure from climate change and may need additional conservation planning. The genetic data also suggest that while species identities are intact, the diversity within these species is impacted by changes in the environment. The spatial analysis in these species can be a useful tool in developing comprehensive conservation strategies that include both *in-situ* and *ex-situ* elements. In combination with the International Treaty on Plant Genetic Resources for Food and Agriculture (ITPGRFA, FAO 2009), these pose the urgent need for the development of specific conservation strategies to consider the effects of climate change. In Article 5 of the ITPGRFA on Conservation, Exploration, Collection, Characterization, Evaluation and Documentation of Plant Genetic Resources for Food and Agriculture^4^, it is reported that each contracting party shall subject to national legislation, and in cooperation with other contracting parties, where appropriate support an integrated approach to promoting *in-situ* conservation of wild crop relatives and wild plants for food production. Predicted current spatial patterns resulting from niche modelling can also contribute to discovering new populations of the target species^55^. Geographical and ecological information has been key to many successful germplasm-collecting missions, as well as to the preservation of extant diversity in *ex-situ* collections, including legumes^49,56^.

## Conclusions

This is the first comprehensive study of wild pea diversity. The analysis utilizing classical phylogenetic markers (ITS and cpDNA) supports that hybridization between wild peas is not an extensive phenomenon. Ecological niche modelling results support that the predicted genetic diversity centers of wild pea in the Mediterranean area may have been driven by Miocene-Pliocene events. These findings set conservation priorities/needs in the implementation of Article 5 of the International Treaty on Plant Genetic Resources for Food and Agriculture and provide geographical and ecological information for germplasm-collecting missions, as well as for the preservation of extant diversity in *ex-situ* collections.

## Methods

### Plant material

Samples for this study were drawn from two discrete, complementary sources. In order to obtain the cp DNA and ITS haplotypes, we used 364 samples of *P*. *sativum* subsp. *elatius* (216) and *P*. *fulvum* (148) (Table S1). We employed the taxonomical classification of Ambrose and Maxted^21^. A subset of 161 samples (143 *P*. *sativum* subsp. *elatius* and 18 *P*. *fulvum*), which included 29 samples from *in-situ* sampling, were used for genome-wide DArTseq (Fig. 1, Table S1) analysis. This material was collected between 1970 and 1990, largely prior to the use of GPS collection site identification. Thus, for some accessions with early collection dates, latitudes and longitudes used in the current study are based on the estimates of passport site description data. These accessions are therefore likely to be less precisely located. The samples were retrieved based on reliable origin^22^, tested for possible duplication (by passport data), cultivated in greenhouses and analyzed for possible misidentification (by morphological assessment of wild traits, namely pod dehiscence, seed dormancy and typical phenotype of wild forms)^57^. Notably, most of the material underwent germplasm multiplication; due to the predominant selfing, it is expected to be highly homozygous and therefore resistant to the effects of genetic drift. Moreover, important herbaria were inspected and samples taken from 109 vouchers (Table S1).

### DNA isolation, PCR amplification and sequence analysis

Genomic DNA used for DArTseq analysis was isolated from single-plant samples. The DArTseq methodology requires high-molecular-weight DNA, typically obtained only from fresh material, while *ITS* and *trnSG* regions were PCR amplified and sequenced; therefore, herbarium samples could be used. PCR reactions were performed, using primers for *ITS* and *trnSG* regions^20,58^. PCR products were treated with Exonuclease-Alkaline Phosphatase (Thermo Scientific) and sequenced (BigDye Terminator v3.1 kit) at Macrogene. Haplotype network analysis was performed with PopART using a median-joining algorithm^59^.

### DArTseq analysis

Genomic DNA was subjected to the standardized next-generation sequencing technique called DArTseq analysis at Diversity Arrays Technology Ltd. using proprietary methodology^60^. Approximately 2,500,000 (+/−7%) sequences per barcode/sample were used for marker calling using DArT PL’s proprietary DArTseq (SNP data) and SilicoDArT (binary presence/absence data) algorithms (DArTsoft14).

### Molecular Data Analysis

Bayesian model-based clustering was performed using STRUCTURE^26,61^, which has been widely used on cultivated and wild pea germplasm^14,24,25^. Population structure was assessed using 161 accessions (*P*. *sativum* subsp. *elatius* & *P*. *fulvum*) with 66,910 polymorphic markers to infer genetic structure and to define the number of clusters using the STRUCTURE software version 2.3.4. The number of presumed populations (K) was evaluated from 3 to 16. The length of the burn-in period was set to 10,000, after which 200,000 iterations of the Monte Carlo Markov Chain (MCMC) were used for data collection. We ran 4 replicate MCMC chains for each value of K to evaluate the posterior likelihood using the ad hoc delta K method^26^. Principal component analysis was performed using the eigen function of R software (R Core Team) after applying a normalization technique^62^. Spatial autocorrelation analysis using SPAGeDi^63^was performed to assess the relationship between individual genetic identities and their geographic distance. We selected samples from Turkey and the Near East only in order to exclude the influence of seas and prohibitively large distances. Ritland´s kinship coefficient^64^ was employed to quantify average pairwise genetic identity based on 20 distance groups in each group with 200 pairwise comparisons. Randomization testing with 100 permutations was conducted to assess whether individual kinship values differed from expectations.The first 15 pairwise comparisons with the highest kinship coefficient from two potentionally interesting distance groups with a mean distance of 617 km and 888 km were depicted using Google Maps (https://maps.google.com/). Pairwise estimation of population Fst was done using the *hierfstat* package in R. The heterozygosity of the detected SNPs within the DArTseq dataset was calculated as a percentage of loci heterozygous per individual. Furthermore, the heterozygosity of putative interspecies hybrids was calculated for sets of SNPs associated (P-value of < 5 × 10^−8^) with respective parental species. To visualize the diversity and structure of the the individual samples in a complementary way, an unrooted split decomposition tree was rendered with the unfiltered DArTsilico data containing 187,298 binary characters using SplitsTree^65^.

### Niche Analysis

Using the location data for 409 *P*. *sativum* subsp. *elatius* and 106* P*. *fulvum* accessions (Table S1), the potential climatic niches were modelled using Maxent version 3.3.3k^66^. Samples that were removed earlier as duplicates, misidentified or otherwise inappropriate, as well as those that had dubious or inaccurate coordinates, were not included in the modelling. A threshold value of 50 km has been used as the maximum accepted distance, and the validation process took place using free available scripts (http://www.movable-type.co.uk/scripts/latlong.html). All the rejected sites have been omitted from the analyses, and validation tests were applied^67^. The environmental predictors used (19 bioclimatic variables)^68^ were from www.worldclim.org. The potential niches of the species were projected in past (Last Glacial Maximum, LGM ~22.000 ybp, http://worldclim.org) and future climatic conditions, following in the latter case the Representative Concentration Pathway (RCP) 6.0 scenario using bioclimatic data created by the Global Climate Model CCSM (Community Climate System Model) 4.0. In order to assess the importance of niche differences between the three species, we performed pairwise niche similarity tests^69^. These tests compare the “observed” niche overlap of the species in question with the “expected” overlap based on the species’ environmental backgrounds. The “observed” overlap, calculated using the metrics D and I, refers to the overlap of the species’ potential niches as they were estimated by Maxent^70^. The “expected” overlap results from substituting the species’ occurrence points with random points from their backgrounds and from calculating D and I for the resulting species/background pair. This random substitution process is iterated a set number of times (100 in our case) in order to obtain a statistical distribution for the two overlapping metrics, against which the “observed” values are tested. The background for each species was derived from its actual occurrence points using a Gaussian filter^67^. Niche similarity tests were performed in ENMTools version 1.4.3^71^. Niche diversity among species, as well as their genotypic groups, was investigated with the use of Shannon’s index of diversity. Typically, this index is expressed as

$${\rm{H}}^{\prime} ={\sum }_{i=1}^{{\rm{R}}}{{\rm{p}}}_{{\rm{i}}}{{\rm{lnp}}}_{{\rm{i}}},$$

where *H′* is Shannon’s diversity index, and *p*_*i*_ is the proportion of individuals (or cover) of the *i*th species in the dataset of interest. In our case, *p*_*i*_ is the probability of occurrence of the *i*th species, and thus *H′* can be calculated on a per-cell basis. The index has been calculated separately for the species using the modelling results of each taxon, as well as for the cpDNA haplotypes that were found during the genetic analysis, using the modelling results of each haplotype. Our quantitative analysis is one of the first to apply Shannon’s diversity index with probabilities of Maxent output to a niche modelling approach^72^. The index was calculated for each cell of the study area using a custom R script. For the manipulation and plotting of spatial data, as well as for the creation of figures, the packages sp, SDMTools and plotrix were employed^73–75^.

### Data availability

Sequences of ITS and trnSG regions were deposited in the NCBI database, and accession numbers are listed in Table S1.

## Electronic supplementary material

Supplementary Information

Dataset S1

## References

[1] Smýkal P, Coyne C, Ambrose MJ (2015). Legume crops phylogeny and genetic diversity for science and breeding. Crit. Rev. Plant Sci..

[2] Foyer CH, Lam HM, Nguyen HT (2016). Neglecting legumes has compromised human health and sustainable food production. Nature Plants.

[3] Godfray HC (2010). Food security: the challenge of feeding 9 billion people. Science.

[4] International Treaty on Plant Genetic Resources for Food and Agriculture, FAO, Roma, Italy (2009)

[5] Vincent H (2013). A prioritized crop wild relative inventory to help underpin global food security. Biol. Conserv..

[6] McCouch S, Baute GJ, Bradeen J (2013). Agriculture: feeding the future. Nature.

[7] Dempewolf H (2014). agriculture to climate change: a global initiative to collect, conserve, and use crop wild relatives. Agroecol.Sust.Food Systems.

[8] Ellis, T. H. N. *Wild Crop Relatives*, *Genomic and Breeding Resources(ed*. *Kole C*.*)*, *237–248 (Berlin-Heidelberg*, *Springer-Verlag 2011)*.

[9] Castañeda-Álvarez NP, Khoury CK, Achicanoy HA (2016). Global conservation priorities for crop wild relatives. Nature Plants.

[10] Ford-Lloyd BV, Schmidt M, Armstrong SJ (2001). Crop wild relatives - undervalued, underutilized and under threat?. BioScience.

[11] Hajjar R, Hodgkin T (2007). The use of wild relatives in crop improvement: a survey of developments over the last 20 years. Euphytica.

[12] Smýkal PPea (2014). *Pisum sativum* L.) in biology prior and after Mendel’s discovery. Czech J: Genet. Plant Breed..

[13] Abbo S, Lev-Yadun S, &Gopher A (2010). Agricultural origins, centers and noncenters; a Near Eastern reappraisal. Crit. Rev. Plant Sci..

[14] Smýkal P, Kenicer G, Flavell A (2011). Phylogeny, phylogeography and genetic diversity of the *Pisum* genus. Plant Genet.Res..

[15] De Candolle, A. *Origin of cultivated plant*s. (1884) Whitefish, Kessinger Publishing (2006).

[16] Vavilov NI (1951). The origin, variation, immunity and breeding of cultivated plants.Translated from the Russian by K. Starchester. Chronica Botanica1.

[17] Kislev ME, Bar-Yosef O (1988). The Legumes, The Earliest Domesticated Plants in the Near East?. Curr.Anthrol..

[18] Smartt, J. *Grain Legumes*, *Evolution and Genetic Resources*. Cambridge, Cambridge University Press (1990).

[19] Zohary, D. & Hopf, M. *Domestication of Plants in the Old World*. Oxford University Press, Oxford.(2000).

[20] Schaefer H, Hechenleitner P, Santos-Guerra A (2012). Systematics, biogeography, and character evolution of the legume tribe Fabeae with special focus on the middle-Atlantic island lineages. BMC Evol.Biol..

[21] Maxted, N. & Ambrose, M. Peas (*Pisum* L.). In, Maxted N., & Bennett, S.J., eds *Plant Genetic Resources of Legumes in the Mediterranean*.Dordrecht, Kluwer Academic Publishers, 181–190 (2001).

[22] Smýkal, P., Coyne, C., Redden R. & Maxted, N. Peas. In, Singh, M., Upadhyaya,H.D., Bisht, I.S., eds. *Genetic and Genomic Resources of Grain Legume Improvement*. Amsterdam, Elsevier (2013).

[23] Jing R, Johnson R, Seres A (2007). Gene-based sequence diversity analysis of field pea (*Pisum*). Genetics.

[24] Jing R, Vershinin A, Grzebyta J (2010). The genetic diversity and evolution of field pea (*Pisum*) studied by high throughput retrotransposon based insertion polymorphism (RBIP) marker analysis. BMC Evol.Biol..

[25] Holdsworth WL, Gazave E, Cheng P (2017). A community resource for exploring and utilizing genetic diversity in the USDA pea single plant plus collection. Hort. Res..

[26] Earl DA, von Holdt BM (2012). STRUCTURE HARVESTER, a website and program for visualizing STRUCTURE output and implementing the Evanno method. Conserv.Genet.Resources.

[27] Warschefsky E, Penmetsa RV, Cook DR, von Wettberg EJ (2014). Back to the wilds, Tapping evolutionary adaptations for resilient crops through systematic hybridization with crop wild relatives. Am. J. Bot..

[28] Palmer JD, Jorgensen RA, Thompson WF (1985). Chloroplast DNA variation and evolution in *Pisum*, Patterns of change and phylogenetic analysis. Genetics.

[29] Polans NO, Moreno RR (2009). Microsatellite and ITS sequence variation in wild species and cultivars of pea. Pisum Genet..

[30] Kosterin OE, Bogdanova VS (2008). Relationship of wild and cultivated forms of *Pisum* L. as inferred from an analysis of three markers, of the plastid, mitochondrial and nuclear genomes. Genet.Resour. Crop Evol..

[31] Kujur A, Bajaj D, Upadhyaya HD (2015). Employing genome-wide SNP discovery and genotyping strategy to extrapolate the natural allelic diversity and domestication patterns in chickpea. Front. Plant Sci..

[32] van Oss R (2015). Genetic relationship in *Cicer sp*. expose evidence for geneflow between the cultigen and its wild progenitor. PLoS ONE.

[33] Saxena RK, von Wettberg E, Upadhyaya HD (2014). Genetic diversity and demographic history of *Cajanus spp*. illustrated from genome-wide SNPs. PLoS ONE.

[34] Rodriguez M (2015). Landscape genetics, adaptive diversity and population structure in *Phaseolus vulgaris*. New Phytol..

[35] Zhou Z, Yu Jiang Y, Wang Z, Gou Z (2015). Resequencing 302 wild and cultivated accessions identifies genes related to domestication and improvement in soybean. Nature Biotech..

[36] Wang J (2016). Development and application of a novel genome-wide SNP array reveals domestication history in soybean. Sci. Rep..

[37] Ladizinsky, G., Abbo, S. The search for wild relatives of cool season legumes.The *Pisum* genus.pp. 55-71 (Springer 2015).

[38] Desiderio F (2013). Chloroplast microsatellite diversity in *Phaseolus vulgaris*. Front. Plant Sci..

[39] Kim, K., Lee, S. C., Lee, J., Yu, Y. *et al*. Complete chloroplast and ribosomal sequences for 30 accessions elucidate evolution of *Oryza* AA genome species. *Sci Rep* **5**, 15655, 10.1038/srep15655. 10.1038/srep15655 PMC4623524 26506948

[40] Vershinin AV, Allnutt TR, Knox MR, Ambrose MJ, Ellis THN (2003). Transposable elements reveal the impact of introgression, rather than transposition, in *Pisum* diversity, evolution, and domestication. Mol. Biol. Evol..

[41] Bogdanova VS, Kosterin OE (2007). Hybridization barrier between *Pisum fulvum* Sibth.et Smith and *P*. *sativum* L. is partly due to nuclear-chloroplast incompatibility. *Pisum*. Genetics.

[42] Raven, P. H. & Polhill, R. M. Biogeography of the Leguminosae. In: Polhill, R. M. & Raven P. H., eds *Advances in Legume Systematics* Kew, RBG, 27–34 (1981).

[43] Schrire BD, Lavin M, Lewis GP (2005). Global distribution patterns of the Leguminosae: insights from recent phylogenies. BiologiskeSkrifter.

[44] Axelrod DI (1975). Evolution and biogeography of Madrean-Tethyansclerophyll vegetation. Ann. Missouri Bot. Garden.

[45] Fernández-Palacios JM (2011). A reconstruction of Palaeo-Macaronesia, with particular reference to the long-term biogeography of the Atlantic island laurel forests. J. Biogeog..

[46] Toussaint-Samat, M. A History of Food. John Wiley & Sons (2009).

[47] Nieto Feliner G (2014). Patterns and processes in plant phylogeography in the Mediterranean Basin. A review. Persp. Plant Ecol. Evol. Syst..

[48] Kropf M, Kadereit JW, Comes HP (2002). Late Quaternary distributional stasis in the submediterranean mountain plant *Anthyllismontana* L. (Fabaceae) inferred from ITS sequences and amplified fragment length polymorphism markers. Mol. Ecol..

[49] Russell J (2014). Genetic Diversity and Ecological Niche Modelling of Wild Barley: Refugia, Large-Scale Post-LGM Range Expansion and Limited Mid-Future Climate Threats?. PLoS ONE.

[50] Fady B, Conord C (2010). Macroecological patterns of species and genetic diversity in vascular plants of the Mediterranean basin. Diver.Distr..

[51] Casimiro-Soriguer R (2010). Phylogeny and genetic structure of *Erophaca* (Leguminosae), a East–West Mediterranean disjunct genus from the Tertiary. Mol. Phyl. Evol..

[52] Manafzadeh, S., Staedler, Y. M. & Conti, E. Visions of the past and dreams of the future in the Orient: the Irano‐Turanian region from classical botany to evolutionary studies. *Biol*. *Rev*, 10.1111/brv.12287 (2016). 10.1111/brv.12287 27349491

[53] Coyne C. J. *et al*. Chapter 8. Genetic Adjustment to Changing Climates: Pea In: S. S. Yadav, B. Redden, J. L. Hatfield, H. Lotze-Campen, Editors. Crop Adaptation to Climate Change.Wiley-Blackwell, Ames, IA. pp. 238–249 (2011).

[54] Bena G, Lyet A, Huguet T, Olivier I (2005). *Medicago–Sinorhizobiu*m symbiotic specificity evolution and the geographic expansion of *Medicag*o. J. Evol. Biol..

[55] Guisan A (2006). Using niche‐based models to improve the sampling of rare species. Conserv.Biol..

[56] Ramirez-Villegas J, Khoury C, Jarvis A, Debouck DG, Guarino L (2010). A gap analysis methodology for collecting crop gene pools: A case study with *Phaseolus* beans. PLoS ONE.

[57] Pavelková A, Moravec J, Hájek D, Baren I, Sehnalová J (1986). Descriptor list genus *Pisum* L. RICP Prague. GenovéZdroje.

[58] Smýkal P (2008). Genetic diversity and population structure of pea (*Pisum sativum* L.) varieties derived from combined retrotransposon, microsatellite and morphological marker analysis. Theor. Appl. Genet..

[59] Bandelt H, Forster P, Röhl A (1999). Median-joining networks for inferring intraspecific phylogenies. Mol. Biol. Evol..

[60] Kilian A, Wenzl P, Huttner E (2012). Diversity Arrays Technology, a generic genome profiling technology on open platforms. Methods Mol. Biol..

[61] Pritchard JK, Stephens M, Donnelly PJ (2000). Inference of population structure using multilocus genotype data. Genetics.

[62] Patterson N, Price AL, Reich D (2006). Population Structure and Eigenanalysis. PLoS Genetic..

[63] Hardy OJ, Vekemans X (2002). SPAGeDi: a versatile computer program to analyse spatial genetic structure at the individual or population levels. Mol. Ecol. Notes.

[64] Ritland K (1996). Estimators for pairwise relatedness and individual inbreeding coefficients. Genet Res..

[65] Huson DH, Bryant D (2006). Application of Phylogenetic Networks in Evolutionary Studies. Mol. Biol. Evol..

[66] Phillips SJ, Anderson RP, Schapire RE (2006). Maximum entropy modeling of species geographic distributions. Ecol. Model..

[67] Alsos IG, Alm T, Normand S, Brochmann C (2009). Past and future range shifts and loss of diversity in dwarf willow (*Salix herbacea* L.) inferred from genetics, fossils and modelling. Glob.Ecol. Biog..

[68] Hijmans RJ, Cameron SE, Parra JL, Jones PG, Jarvis A (2005). Very high resolution interpolated climate surfaces for global land areas. Inter. J. Climatol..

[69] Warren DL, Glor RE, Turelli M (2008). Environmental niche equivalency versus conservatism, quantitative approaches to niche evolution. Evolution.

[70] Allouche O, Steinitz O, Rotem D, Rosenfeld A, Kadmon R (2008). Incorporating distance constraints into species distribution models. J. Appl. Ecol..

[71] Warren DL, Glor RE, Turelli M (2010). ENMTools, a toolbox for comparative studies of environmental niche models. Ecography.

[72] Vorsino AE (2014). Modeling Hawaiian ecosystem degradation due to invasive plants under current and future climates. PLoS One.

[73] Pebesma EJ, Bivand RS (2005). Classes and methods for spatial data in R. R News.

[74] Van Der Wal, J., Falconi, L., Januchowski, S., Shoo, L. & Storlie, C. SDMTools, Species Distribution Modelling Tools, Tools for processing data associated with species distribution modelling exercises. http,//CRAN.R-project.org/package=SDMTools. (2014).

[75] Lemon J, Plotrix a (2006). package in the red light district of R. R News.

