# Author Correction: Genomic diversity and macroecology of the crop wild relatives of domesticated pea

**DOI:** 10.1038/s41598-018-30987-5

**Published:** 2018-08-29

**Authors:** Petr Smýkal, Iveta Hradilová, Oldřich Trněný, Jan Brus, Abhishek Rathore, Michael Bariotakis, Roma Rani Das, Debjyoti Bhattacharyya, Christopher Richards, Clarice J. Coyne, Stergios Pirintsos

**Affiliations:** 1 Department of Botany, Palacký University in Olomouc, Olomouc, Czech Republic; 2 Agricultural Research, Ltd., Troubsko, Czech Republic; 3 Department of Geoinformatics, Palacký University in Olomouc, Olomouc, Czech Republic; 4 The International Crops Research Institute for the Semi-Arid Tropics, Hyderabad, India; 5 Department of Biology and Botanical Garden, University of Crete, Heraklion, Greece; 6 Department of Life Science & Bioinformatics, Assam University, Silchar, India; 7 United States Department of Agriculture, National Laboratory for Genetic Resources Preservation, Fort Collins, USA; 8 United States Department of Agriculture, Washington State University, Pullman, Washington USA

Correction to: *Scientific Reports* 10.1038/s41598-017-17623-4, published online 12 December 2017

In the Supplementary Information file ‘Dataset S1’ originally published with this Article, in the ‘DARTseq-samples’ tab, sample WL2140 was incorrectly labelled as JI2140. Furthermore, samples 711, 713, 714, 721, 722, P014, P016, P017, and L100 were incorrectly labelled in the ‘P_sativum-elatius’ and ‘DARTseq-samples’ tabs as originating from VIR, Russia. The label now reads ‘Novosibirsk, Russia’.

These errors have been corrected in the Supplementary Information that now accompanies the Article.

