# Supplementary material for: Genomic diversity and macroecology of the crop wild relatives of domesticated pea
Source: Sci Rep. 2017 Dec 12;7:17384. doi: 10.1038/s41598-017-17623-4 (PMC5727218; doi:10.1038/s41598-017-17623-4)
Supplement: Supplementary file 1 — Supplementary Information [file 41598_2017_17623_MOESM1_ESM.pdf]

**Genomic diversity and macroecology of the crop wild relatives of domesticated pea**

Petr Smýkal[1*], Iveta Hradilová[1], Oldřich Trněný[2], Jan Brus[3], Abhishek Rathore[4], Michael Bariotakis[5], Roma Rani Das[4], Debjyoti Bhattacharyya[6], Christopher Richards[7], Clarice J. Coyne[8], Stergios Pirintsos[5]

**Figure S1**

Evannno delta K indicating optimal number of clusters (K=6)

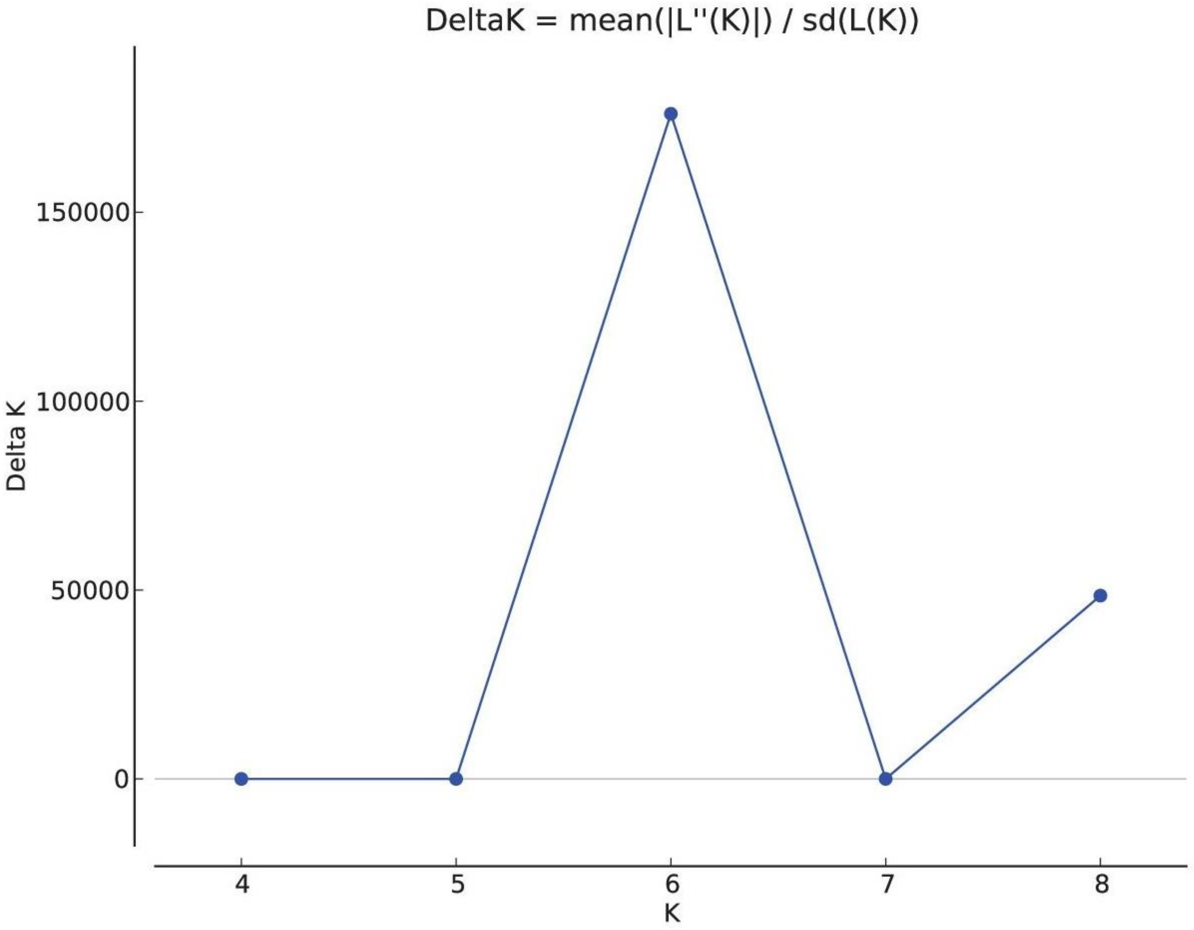

Inferred population structure of wild *Pisum sp.* for the K values 3 to 7.

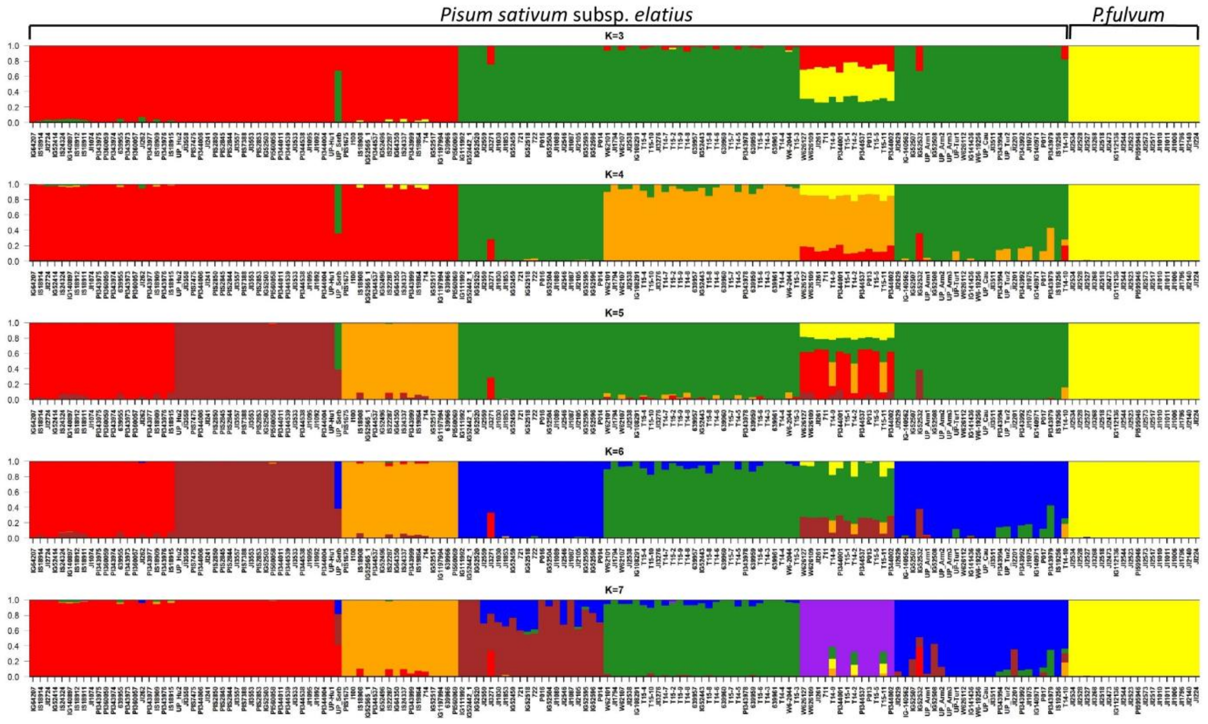


**Figure S2**

Results of niche similarity tests between *Pisum sativum* subsp. *elatius* complex and *P. fulvum* based on Relative Rank. The green bars correspond to simulations of similarity between *Pisum sativum* subsp. *elatius* complex and points drawn from the background of *P. fulvum*, while the blue bars correspond to the reverse simulations. The red vertical line shows the observed value of the Relative Rank for the two species. The numbers on the upper left corner represent the p-values of the respective tests. Histograms of niche similarity tests results for the species *P. fulvum* and *P. sativum* subsp. *elatius*. Each plot represents the pair-wise test between two species, the names of which can be derived from the diagonal. The plots above the diagonal correspond to niche overlap metric D, while those below the diagonal correspond to metric I. In each plot, two distributions of values can be found, each corresponding to the test of the species labeled with the same colour against the background of the

other species. The bold, vertical lines show the observed niche overlap for each species pair (created with R version 3.2.2. https://cran.r-project.org/bin/windows/base/old/3.2.2/).

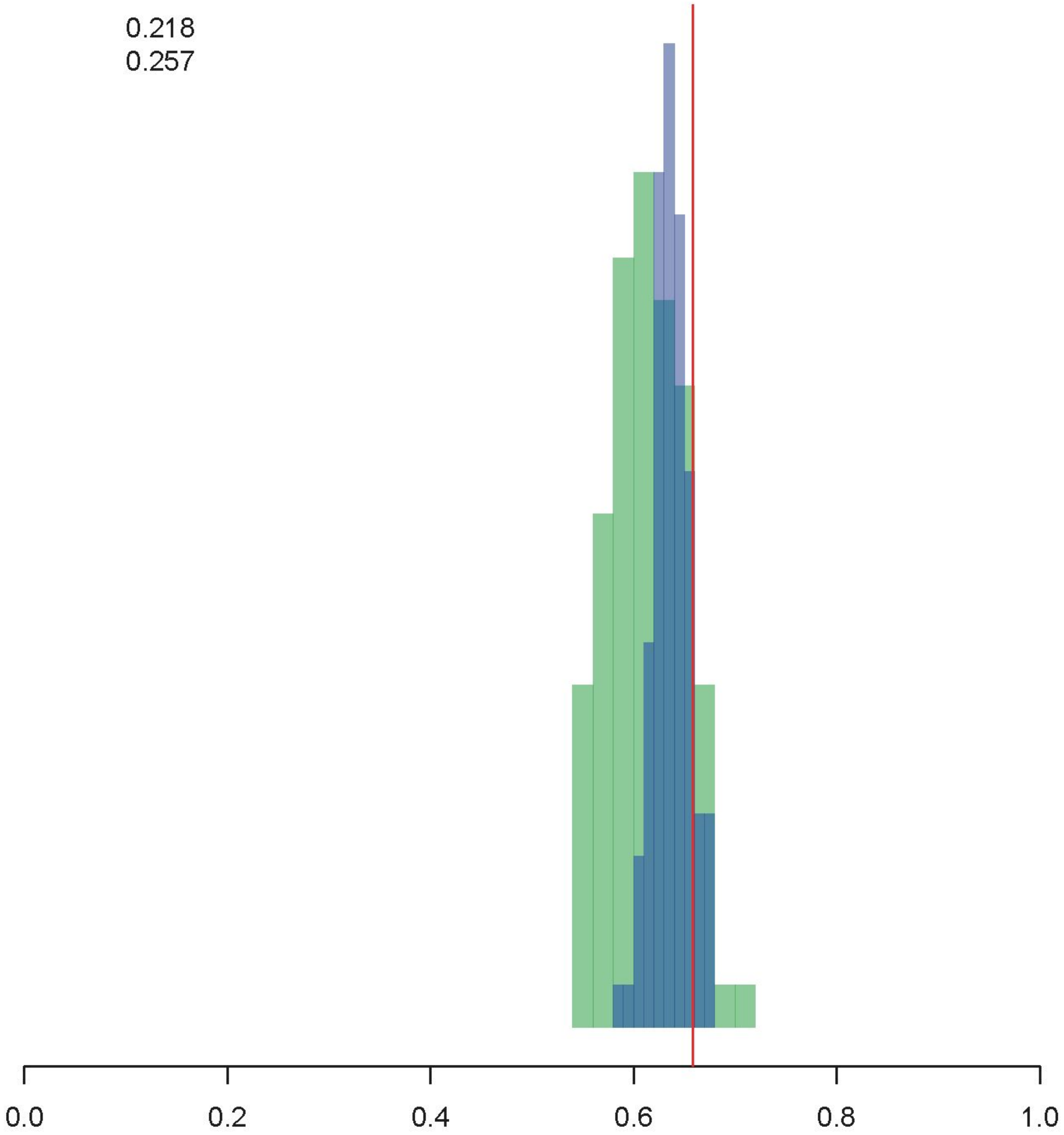

**Figure S3**

Results of pairwise niche similarity tests between the cpDNA haplotypes, E1, E2, E3, E5, E6 and F based on Relative Rank. The red vertical lines show the observed values of the Relative Rank in each case. The numbers on the upper left corner represent the p-values of the respective tests (created with R version 3.2.2. https://cran.r-project.org/bin/windows/base/old/3.2.2/).

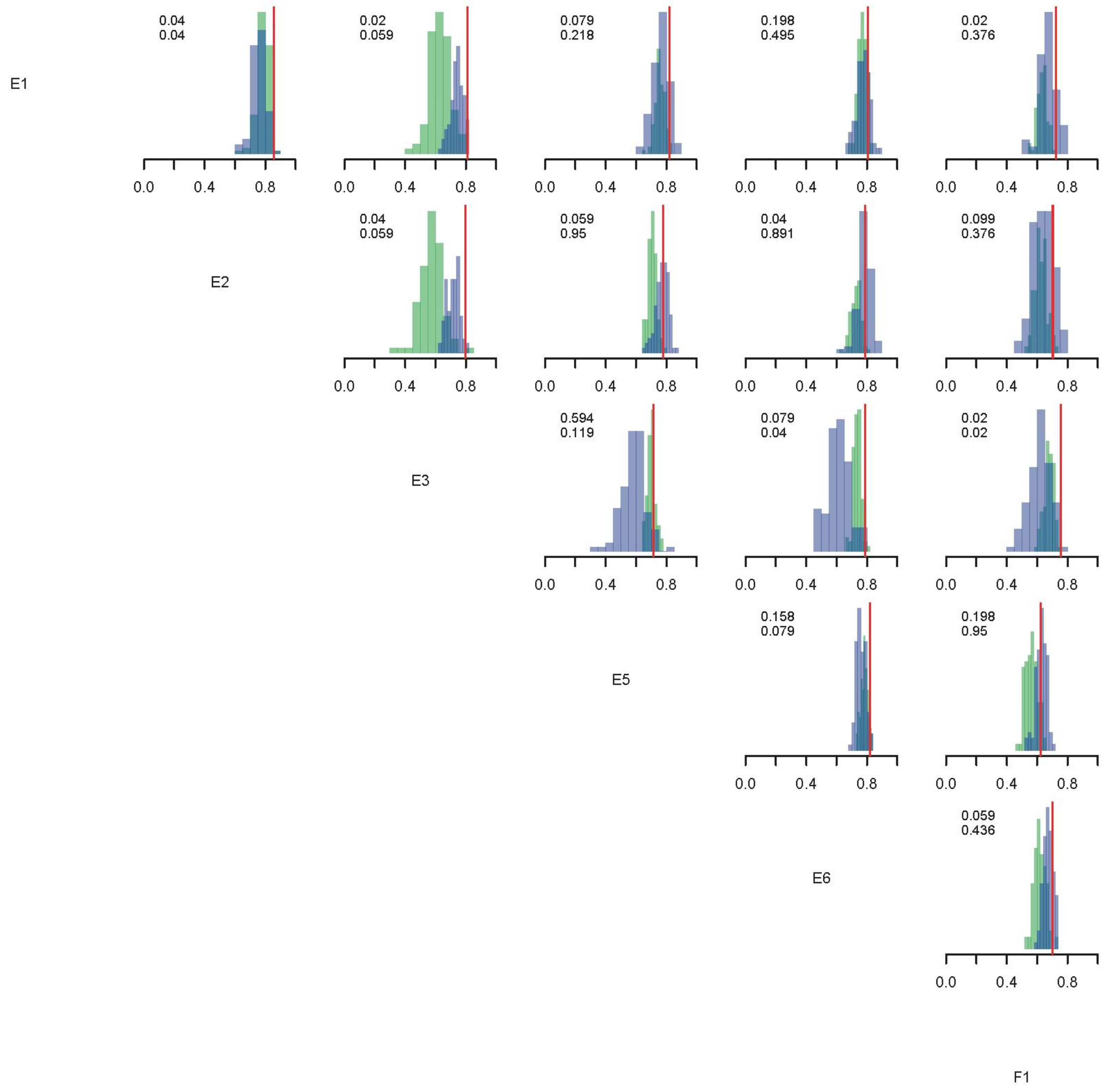

# Table S2

Summary of detected cpDNA and ITS haplotypes.

| | trnSG haplotypes | | | | | | | |
|---|---|---|---|---|---|---|---|---|
| | F1 | E1 | E2 | E3 | E4 | E5 | E6 | Total |
| ***P. sativum* subsp. *elatius*** | | 34 | 29 | 21 | 5 | 68 | 58 | 215 |
| ***P. fulvum*** | 143 | | | 1 | | | 5 | 149 |
| | | | | | | | | 364 |

| | ITS haplotypes | | | | | | | | | | | | | | |
|---|---|---|---|---|---|---|---|---|---|---|---|---|---|---|---|
| | *f1* | *f2* | *f3* | *f4* | *ela1* | *ela2* | *ela3* | *ela4* | *ela5* | *ela6* | *ela7* | *ela8* | *ela9* | *ela10* | *ela11* |
| ***P. sativum* subsp. *elatius*** | 1 | | | | 27 | 6 | 10 | 19 | 13 | 6 | 12 | 3 | 1 | 5 | 3 |
| ***P. fulvum*** | 72 | 4 | 23 | 49 | | | | | | | | | | | |

| | *ela12* | *ela13* | *ela14* | *ela15* | *ela16* | *ela17* | *ela18* | *ela19* | *ela20* | *ela21* | *ela22* | *ela23* | *ela24* | *unique* |
|---|---|---|---|---|---|---|---|---|---|---|---|---|---|---|
| ***P. sativum* subsp. *elatius*** | 11 | 41 | 9 | 9 | 6 | 2 | 1 | 2 | 2 | 4 | 3 | 3 | 2 | 17 |
| ***P. fulvum*** | | | | | | | | | | | | | | |
